# Phylogenetic position of the acariform mites: sensitivity to homology assessment under total evidence

**DOI:** 10.1186/1471-2148-10-235

**Published:** 2010-08-02

**Authors:** Almir R Pepato, Carlos EF da Rocha, Jason A Dunlop

**Affiliations:** 1 Departamento de Zoologia, Instituto de Biociências, Universidade de São Paulo, Rua do Matão, travessa 14, 321, 05508-900, São Paulo, Brazil; 2 Museum für Naturkunde, Leibniz Institute for Research on Evolution and Biodiversity at the Humboldt University Berlin, Invalidenstrasse 43, 10115 Berlin, Germany

## Abstract

**Background:**

Mites (Acari) have traditionally been treated as monophyletic, albeit composed of two major lineages: Acariformes and Parasitiformes. Yet recent studies based on morphology, molecular data, or combinations thereof, have increasingly drawn their monophyly into question. Furthermore, the usually basal (molecular) position of one or both mite lineages among the chelicerates is in conflict to their morphology, and to the widely accepted view that mites are close relatives of Ricinulei.

**Results:**

The phylogenetic position of the acariform mites is examined through employing SSU, partial LSU sequences, and morphology from 91 chelicerate extant terminals (forty Acariformes). In a static homology framework, molecular sequences were aligned using their secondary structure as guide, whereby regions of ambiguous alignment were discarded, and pre-aligned sequences analyzed under parsimony and different mixed models in a Bayesian inference. Parsimony and Bayesian analyses led to trees largely congruent concerning infra-ordinal, well-supported branches, but with low support for inter-ordinal relationships. An exception is Solifugae + Acariformes (P. P = 100%, J. = 0.91). In a dynamic homology framework, two analyses were run: a standard POY analysis and an analysis constrained by secondary structure. Both analyses led to largely congruent trees; supporting a (Palpigradi (Solifugae Acariformes)) clade and Ricinulei as sister group of Tetrapulmonata with the topology (Ricinulei (Amblypygi (Uropygi Araneae))). Combined analysis with two different morphological data matrices were run in order to evaluate the impact of constraining the analysis on the recovered topology when employing secondary structure as a guide for homology establishment. The constrained combined analysis yielded two topologies similar to the exclusively molecular analysis for both morphological matrices, except for the recovery of Pedipalpi instead of the (Uropygi Araneae) clade. The standard (direct optimization) POY analysis, however, led to the recovery of trees differing in the absence of the otherwise well-supported group Solifugae + Acariformes.

**Conclusions:**

Previous studies combining ribosomal sequences and morphology often recovered topologies similar to purely morphological analyses of Chelicerata. The apparent stability of certain clades not recovered here, like Haplocnemata and Acari, is regarded as a byproduct of the way the molecular homology was previously established using the instrumentalist approach implemented in POY. Constraining the analysis by *a priori *homology assessment is defended here as a way of maintaining the severity of the test when adding new data to the analysis. Although the strength of the method advocated here is keeping phylogenetic information from regions usually discarded in an exclusively static homology framework; it still has the inconvenience of being uninformative on the effect of alignment ambiguity on resampling methods of clade support estimation. Finally, putative morphological apomorphies of Solifugae + Acariformes are the reduction of the proximal cheliceral podomere, medial abutting of the leg coxae, loss of sperm nuclear membrane, and presence of differentiated germinative and secretory regions in the testis delivering their products into a common lumen.

## Background

Acari (mites and ticks) have been variously ranked as a group composed of one to seven or more distinct orders [1]. Together they comprise approximately half of the described arachnid diversity [2]. Two main lineages are traditionally recognized: Acariformes (or Actinotrichida) and Parasitiformes (or Anactinotrichida). Although Opiloacariformes has been regarded as a third, distinct order [3], both internal and external morphology leaves little doubt that they should be included within the Parasitiformes [4-6].

Of the two main lineages, Acariformes is the most diverse and comprises around two thirds of the known species of mites [2]. It is also an ancient group including representatives from the two of the earliest terrestrial invertebrate communities: the Rhynie Chert (Scotland) and the Gilboa Formation (New York State, USA), from the early and mid Devonian respectively. By contrast Parasitiformes appears in the fossil record only in the Mesozoic era [7] and is represented by far fewer fossil species. Among modern Acariformes, a bewildering array of lifestyles and habitats may be found and the group includes important agricultural pests, plant disease vectors, and animal parasites.

Masta and colleagues [8] explored the use of the mitochondrial genome in inferring arachnid phylogeny, but could employ data from only six of the twelve extant orders. Most of previous studies which explored chelicerate relationships included data from all orders and employed as molecular markers the nuclear ribosomal Small and Large Subunits genes (SSU and LSU rRNA, respectively) [9-11].

Initial work on the internal relationships of Acarifomes performed by one of us revealed that inclusion of many new ribosomal sequences from different acariform mites led to important changes in the topology recovered. In fact, although we agree that more genes must be included in future analysis, reducing sampling biases due to a scarcity of characters (a goal which we are pursuing), we are of the opinion that Acariformes have been largely underrepresented in previous analyses. This, together with ongoing questions about the sister group of mites, motivated the present study. Besides sampling effort, we explored the behavior of the new molecular data when analyzed alone and combined with different morphological matrices and under different analytical approaches. The aim of this was to explore possible drawbacks in the homology establishment for molecular data in previous studies.

### Previous studies on arachnid phylogeny and the position of acariform mites

Weygoldt and Paulus [12] first applied the Hennigean method to arachnid phylogeny and resolved mites as the sister group of Ricinulei. They did not, however, attempt to test the monophyly of Acari since they employed the approach of coding assumed ancestral states for mites instead of scoring Acari in-group polymorphisms.

Lindquist [4] endorsed the notion of a monophyletic Acari by proposing eleven putative shared apomorphies for the clade; nevertheless most of these are mite-specific, 'tendencies' or related to size reduction [6]. Presence of a gnathosoma - i.e. a pseudotagma that includes the mouthparts - and hexapodal larvae were suggested as the main synapomorphies uniting Acari and Ricinulei.

Hammen [13,14] regarded the presence of a gnathosoma in Acari and Ricinulei as non-homologous based on details of gnathosoma morphology; particularly the insertion positions of its musculature. Acariformes was hypothesized to be sister group of Palpigradi (together forming his Epimerata group) and Anactinothrichida the sister group of Ricinulei (his Cryptognomae group). Even though Hammen performed an extensive survey of mite and arachnid morphology, his rejection of cladistics and his controversial scenario of leg coxa evolution in support of Epimerata have lessened the impact of his conclusions.

The phylogenetic analysis performed by Shultz [15,16] again recovered mites as sister group of Ricinulei. This clade was formally named Acaromorpha, although Dubinin [17] coined this name earlier, referring to mites only. Shultz [15,16] performed his analyses after an extensive survey of characters - particularly those relating to appendage musculature - but like Weygoldt and Paulus [12] used supraspecific terminal taxa to code characters. Therefore, he did not test mite monophyly. Nevertheless, the notion of a monophyletic Acari and its inclusion in Acaromorpha *sensu *Shultz has received increasing acceptance among the acarological and arachnological communities. However, it should be reiterated that no new evidence for this clade in terms of explicit synapomorphies has been brought to light since the summary of Lindquist [4].

Wheeler and Hayashi [9] analyzed partial SSU and LSU rRNA sequences from species belonging to all arachnid orders except Palpigradi and recovered a diphyletic Acari in their molecular analysis. Whereas the single Acariformes species included, *Tetranychus urticae*, was recovered in an unlikely position at the base of the cladogram outside the other Chelicerata, Parasitiformes emerged in this analysis as the sister group of Pycnogonida (sea spiders). One should bear in mind, that this analysis must be regarded as a first attempt, including few terminals. No molecular data on the order Palpigradi, for example, could be included. When combining morphological and molecular data a cladogram quite similar to that obtained by Shultz's [16] morphological analysis was recovered; the 'total evidence' analysis differing primarily from Shultz's results in its placement of Amblypygi (whip spiders).

The Acaromorpha hypothesis has been challenged by observations from paleontology and ultrastructure. Dunlop [18], supplemented by Dunlop *et al. *[19], presented a set of putative apomorphies linking Ricinulei to the fossil order Trigonotarbida, which would imply that the presence of a "gnathosoma" in Ricinulei is homoplastic. An analogous scenario had been already proposed by Hammen [20].

Alberti and Peretti [21] confirmed previous observations on Solifugae sperm cells (absence of a carioteca in mature spermatozoa) and on testis structure (presence of differentiated germinative and secretory regions delivering their products into a common lumen). They argued that these observations should be considered as putative apomorphies shared between Solifugae and Acariformes to the exclusion of the Parasitiformes mites.

Giribet *et al. *[10] employed a broader taxon sampling than Wheeler and Hayashi [9], including molecular data from all arachnid orders. In addition, they employed a species exemplar approach for coding the morphological characters, reflecting in the analysis the morphological diversity of the orders, which allows at the same time a test of their monophyly. In the molecular analysis, mites again appear as diphyletic, with Acariformes as a basal group and Parasitiformes here as the sister group of Pseudoscorpiones. The combined analysis of neontological data recovered a monophyletic Acari, but resolved them as a basal lineage far from Ricinulei. When adding fossils and rooting the tree on Trilobita, Ricinulei formed a clade with the fossil order Trigonotarbida; together as sister group of Tetrapulmonata (Araneae, Uropygi and Amblypygi). In this analysis, a monophyletic Acari came out as sister-group of Pycnogonida. Note, however, that both the neontological and paleontological trees are quite similar. If the paleontological tree was rooted on Pycnogonida the resulting topology would be similar to that obtained for the neontological data alone; although with Trilobita as the sister group of Xiphosura.

In an article focusing on Parasitiformes phylogeny, Klompen *et al. *[11] also included seven Acariformes species: three Prostigmata, three Oribatida and one Endeostigmata. Klompen *et al. *[11] employed a methodology completely different from previous molecular studies. Instead of direct optimization as implemented in the program POY, they used the secondary structure as a guide for hypothesizing the nucleotide homology (alignment) and applied parsimony and Bayesian analyses. The results obtained are well supported for Parasitiformes relationships, but lack resolution for the Arachnida orders. They recovered mites as a monophylum, but resolved no clear hypothesis with respect to the mites' sister-group. The Acaromorpha hypothesis required the addiction of 14 steps to the MPTs recovered in their parsimony analysis.

In his latest arachnid study, Shultz [22] offered a thorough revision of arachnid morphology and employed the species exemplar approach for coding characters, i.e. reflecting taxon polymorphism. Acaromorpha was recovered again, but with low support and, when fossils were included, Acari became diphyletic and Ricinulei were recovered as sister group of Parasitiformes only. Concerning mites' relationships, the results of this analysis should be interpreted with caution. Although Shultz cited the article by Alberti and Peretti [21], he did not take into consideration the main reasons which led them to question the association of Solifugae and Pseudoscorpiones (the clade Haplocnemata): namely their testis and spermatozoa structure. Shultz also misinterpreted Alberti and Peretti [21] when evaluating alternative phylogenetic relationships. He stated that these authors clustered both mite lineages with Solifugae (see his figure three), whereas Alberti and Peretti actually proposed that only Acariformes should be related to Solifugae. Finally, some differences among the two main lineages of mites, such as the condition of the sternal region were not correctly scored (for a summary of these differences we refer the reader to [5,6]).

The position and monophyly of Acari has occasionally been tested in larger studies of arthropod phylogeny in general; although we would caution here that the number of mite taxa included as terminals was usually rather small. Regier et al. [23] produced the most comprehensive and up to date molecular phylogeny for arthropods in general, with 62 genes tested for 80 taxa, whereby acariform and parasitiform mites were both represented here by single exemplars (*Dinothrombium *and *Amblyomma *respectively). Within a monophyletic Arachnida, Parasitiformes was recovered as the sister group of Pseudoscorpiones (as per Giribet et al. [10]) with Opiliones as their outgroup. Acariformes was recovered as the sister group of Palpigradi (essentially Hammen's Epimerata hypothesis [13,14]); this clade being basal with respect to all other arachnids.

Finally, in a recent publication Dabert et al. [24] investigated Acariformes phylogeny using 18 S rDNA and mitochondrial cytochrome oxidase subunit I tested across 142 acariform species, plus 34 outgroups. They recovered Acariformes as monophyletic with the traditional split into Trombidiformes and Sarcoptiformes, and for the sarcoptiform mites they provided molecular support for an emerging hypothesis that Oribatida is paraphyletic with respect to Astigmata - the astigmatans being the sister group of a derived oribatid lineage. Age estimates for splits into the major groups were also calculated; with acariform mites estimated as having their origins in the Silurian (ca. 430 Ma), which is broadly consistent with the fossil record (see above). Acari was again recovered as diphyletic. Parasitiformes resolved as the sister group of Pseudoscorpiones, a similar result to that of Giribet et al. [10] and Regier et al. [23]. Significantly, Acariformes resolved in Dabert et al.'s study as sister group to Solifugae, and with good support. Here, we present further evidence for this hypothesis based on combined morphological and molecular data. We also suggest a formal name for this putative clade encompassing acariform mites and camel spiders.

## Methods

### Taxa sampled

We follow Giribet and Ribera [25] in considering Pycnogonida as an appropriate out-group for rooting the resulting tree. Despite Maxmen *et al*.'s [26] hypothesis for the protocerebral nature of the pycnogonid chelifores and the defence of the traditional view of tritocerebral chelicerae by Bitsch and Bitsch [27], data from gene expression and neuroanatomical studies convincingly demonstrate that chelifores, chelicerae and mandibulate antennae are homologous, deutocerebral elements [28-31]. Despite its long list of autapomorphies, Pycnogonida 'chelifores' are considered here to be true chelicerae, thus providing morphological support for sea spiders' placement as sister-group of Euchelicerata.

**Figure 1:**
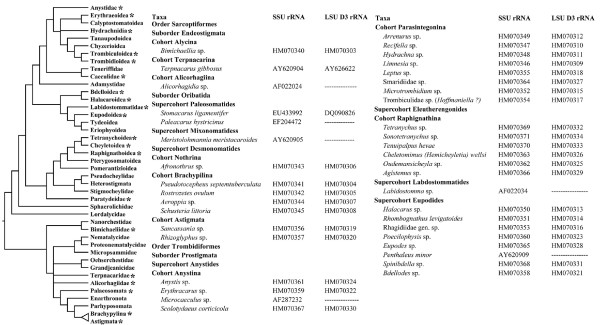
**Acariformes taxa included**. A- Dendrogram representing hypotheses of relationships summarized in the Norton *et al. *[32] study. (*) indicates lineages represented in the present analysis. B- Actinotrichida taxa included in the study.

Representatives from all euchelicerate orders were sampled here, comprising 91 terminal taxa, of which 40 are acariform mites. Among them, one palpigrade, one whip spider, three spiders and 32 actinotrichid mites are newly sequenced. Since a formal cladistic analysis including Acariformes lineages is unavailable, we used the dendrograms summarized in Norton *et al*., [32] as a reference for the sampling design. We tried to include representatives of all major lineages of Acariformes. Table 1 and Fig. 1 summarize the sampling effort. Following the latest account of Acari classification [33], Endeostigmata was retained, although it certainly is not a monophylum [34]. Furthermore, the assignment of the rank Superorder to Acariformes and Parasitiformes is retained, although it certainly does not agree with the current classification of Chelicerata; i.e. 'Superorders' are rarely used for arachnids other than mites.

**Table 1:** Chelicerate non-Actinotrichida included in the analysis

Species	SSU rRNA	LSU D3 rRNA	Species	SSU rRNA	LSU D3 rRNA	Species	SSU rRNA	LSU D3 rRNA
**Class Pycnogonida**			**Order Ricinulei**			*Neobisium polonicum*	EU559357	EU559457
*Achelia echinata*	AF005438	AF005459	*Pseudocellus pearsei *	U91489	AF124956	*Anagarypus heatwolei*	EU559376	EU559482
*Callipallene *sp.	AF005439	AF005460	Ricinoididae sp	AF124930	AF062988	*Americhernes *sp.	AF124934	AF062982
*Endeis laevis*	AF005441	AF005462	**Order Opiliones**			**Order Araneae**		
*Colossendeis *c	AF005440	AF005461	*Siro rubens *	U36998	U91494	*Liphistius bicoloripes *	AF007104	AF124960
**Class Chelicerata**			*Stylocellus *sp.	AF173419	Af173422	*Aphonopelma *sp.	X13457	------------
**Order Xiphosura**			*Odiellus troguloides *	X81441	U91500	*Atypoides riversi*	DQ981699	DQ639855
*Limulus polyphemus *	U91490	U91492	*Pachyloides thorellii*	U37007	U91508	*Nesticus celullanus*	AF005447	AF124961
*Carcinoscorpius rotundicauda*	U91491	U91493	*Caddo agilis*	U91487	U91502	*Lyssomanes viridis *	DQ665742	-----------
**Order Schizomida**			*Sabacon cavicolens*	AF124944	AF124972	Tetragnathidae	HM070337	HM07300
*Stenochrus portoricensis*	AF005444	------------	*Leiobunum *sp.	AF124940	AF124968	Corinnidae	HM070338	HM07301
*Trithyreus pentapeltis*	AF124932	AF062990	*Nemastoma bimaculatum*	AF124947	AF124974	Pholcidae	HM070339	HM07302
**Order Thelyphonida**			*Zuma acuta*	AF124951	AF124978	**Order Solifugae**		
*Mastigoproctus giganteus *	AF005446	AF062989	**Supeorder Parasitiformes**			*Gluvia dorsalis *	AF007103	AF124957
**Order Amblypygi**			*Opilioacarus texanus *	AF124935	AF124963	*Eusimonia wunderlichi*	U29492	AF124958
*Paraphrynus *sp.	AF005445	AF124959	*Amblyomma americanum*	AF291874	AF291874	*Chanbria regalis*	AF124931	AF062983
Amblypygi sp.	AF124933	AF062965	*Otobios megnini*	L76356	-----------	*Eremobates *sp.	AY859573	AY859572
*Musicodamon atlanteus*	AY829903	AY829924	*Allothyrus cf australasiae*	AY620910	AY626628	**Order Scorpiones**		
*Charinus montanus*	HM070335	HM07298	*Sternothyrus braueri*	AY620912	AY626630	*Pandinus imperator *	AY210831	AY156537
**Order Palpigradi**			**Order Pseudoscorpiones**			*Belisarius xambeui *	AF005442	AF124954
*Eukoenenia *n. sp.	AF207648	AF207653	*Chthonius *sp.	EU559387	EU559438	*Androctonus australis *	X77908	AF124955
*Eukonenia *sp.	HM070336	HM07299	*Pseudogarypus bicornis*	EU559368	EU559472			

Species sampled and Genbank accession numbers of non-Acariformes taxa

### DNA extraction, vouchering, amplification and sequencing

Individual body parts, such as a leg article, or entire specimens were used for genomic DNA extraction. When the entire specimen was destroyed by the extraction process, individuals from the same population were kept as vouchers. Voucher material is deposited in the Museu de Zoologia da Universidade de São Paulo (MZSP) and its collection numbers are provided in the additional file 1 along with details on sampling locality.

Small pieces of animal tissue (less than 0.5 mm) for large arachnids or the entire animal for many mites were crushed against the vial wall and mixed with a small water volume. Chelex based solution Instagene^® ^(BIORAD) was added to the vial and incubated for 30 min at 54°C, followed by 8 min at 100°C. The solution was spun and approximately 140 μl of supernatant, in most cases, enough for 14 PCR reactions, was obtained.

The SSU rRNA genes were amplified and sequenced using the three pairs of primers described in Giribet *et al. *[35], or with the intermediary segment pair replaced by the 18SV4F-18SV4R primer designed by Otto & Wilson [36]. For the 28 S D3 region we used the primer pair 28SA-28SB described in Whiting et al. [37].

Amplification was carried out in a 25 μl volume with 0.6 units of Taq Polymerase (Fermentas), 100.00 μM of DNTPs, 2.50 mM of MgCl_2 _and 0.40 μM of each primer. The thermocycler program included an initial denaturing step of 4 min. at 94°C, and 35 amplification cycles of 30 s. of denaturing at 94°C, 30 s of annealing at 50°C, 45s-1 min of extension at 72°C and a final step of extension of 5 min. at 72°C. The PCR products were purified using the Ampure^® ^(Agencourt) kit and sequenced using an ABI Prism 3100 Genetic Analyzer Sequencer. Cycle-sequencing with AmpliTaq DNA polymerase, FS (Perkin-Elmer) using dye-labeled terminators (ABI Prism BigDye Terminator CycleSequencing Ready Reaction Kit) was carried out in a 10 μl volume of reaction: 4 μl of Terminator Ready Reaction Mix, 10-30 ng/ml of PCR product, 5 pmol of primer, and dH2O to 10 μl. The cycle-sequencing program consisted of an initial step at 94°C for 3 min, 25 sequencing cycles (94°C for 10 s, 50°C for 5 s, 60°C for 4 min). The BigDye-labeled PCR products were isopropanol-precipitated following the manufacturer's protocol.

Reverse and direct chromatograms were assembled using the program ChromasPro 1.41 (Technelysium Pty Ltd).

### Analyses

Ribosomal RNA is the core of this organelle's function. It is the target of intense stabilizing selection in order to maintain its catalytical activity. However, this activity is more related to its secondary and tertiary structure, constructed by the correct pairing of RNA nucleotides, than to its nucleotide composition.

Inside stems, a mutation that disturbs the correct base pairing is likely to reduce molecular fitness. A compensatory mutation that re-sets a normal pairing is favored by the selection, replacing one Watson--Crick pairing for another or for slightly less stable guanine and uracile pairs [38]. Pairing between adenine and cytosine is much rarer but may have a similar effect if protonated, since they are geometrically similar to the G:U, U:G pairs [39].

This property of ribosomal gene evolution may therefore be a tool for assessing nucleotide homology. Detection of compensatory or semi-compensatory mutations along a multiple alignment is the main tool employed for inferring ribosomal RNA secondary and tertiary structures [38,40], and has been largely confirmed by crystallographic results [41].

The secondary structure alignment was made employing the method described by Kjer [42], except for employing the program BioEdit 7.0.9 [43] for sequence editing. Template SSU rRNA structures were downloaded from the "European Ribosomal DataBase" [44]. Template LSU structures were obtained from Schnare *et al. *[45]. For both genes, the secondary structures inferred by Rix *et al. *[46] were also valuable.

For regions where the nucleotide composition does not readily allow the detection of correspondence to the models, potential pairings was explored using Mfold [47]. Alignments were produced first for each of the orders using compensatory mutations and similarity as criteria. After this step, common structural motifs were used to align the entire data set. Consensus secondary structures for these alignments were inferred using the program RNAalifold [48]. *Only nucleotides in regions whose alignments were sustained by compensatory mutations across the entire data set were considered as homologous*. Regions inferred to be ambiguously aligned were classified following Gillespie [49] in regions of expansion and contraction (REC), non-pairing regions of ambiguous alignment (RAA) and regions of slipped-strand compensation (RSC). The structural models for exemplar species are presented in the results section using the Wuyts *et al. *[44] notation for SSU rRNA and Cannone *et al. *[50] for LSU. The figures displaying secondary structure features were drawn using the xRNA program (developed by B. Weiser and H. Noller, University of Santa Cruz).

Fasta files containing the alignment labeled with the SSU and LSU rRNA secondary structure are included in the digital supplementary material associated with this article (additional file 2).

### Bayesian phylogenetic inference

Wheeler and Honeycutt [51] demonstrated that stem and loop regions may point to different phylogenies. This is to be expected since compensatory mutations violate the assumption of character independence made by parsimony and most maximum likelihood analyses.

For Bayesian estimation of phylogeny, we employed the program PHASE 2.0 [52] due its inclusion of a great variety of models which encompass base-pairing in stems [53]. Testing each of these models is beyond to the scope of the present study. The aim here is to verify the impact of relaxing the character independence assumption among stem nucleotides on the topology recovered. Hence, the following models were tested using Bayes factors as criteria [54]: (a) A uniform "4by4" nucleotide model, GTR +I +G, a choice made with the assistance of the jMODELTEST program [55]; (b) Distinct GTR +I +G models were employed for stems and loops; (c) GTR +I +G model for loops and a 7A for stems. The 7A model is the most general reversible 7-state model, i.e. one model the base-pair states A:U, U:A, G:C, C:G, G:U, and UG were assumed to be matches and all other base-pairs were lumped in a single mismatch MM state. The model includes 26 free parameters and allows base pair reversal asymmetry, an apparently biologically sound property of the model [56]; (d) GTR +I +G model was assigned to loops and a 16A model to stems. A general time reversible 16 state model would include 134 free parameters, which reduces its utility to real data. In the 16A model, it is simplified to include only 19 free parameters. There are three α_ij _parameters for the six main states, modeling simple substitutions, double substitutions and double transversions; a single parameter for mutations to and from mismatch states and a parameter for single mutations between mismatch states.

Flat priors were used for all analyses. Four Markov chains were used in three runs of the same analysis starting from randomly built trees. At least eight million generations were run to ensure that sampling adequately explored the parameter space. The degree of convergence in tree topologies, clade posterior probabilities and parameter posterior probabilities across all analyses were analyzed in the program TRACER ver. 1.4.1 [57], which provides graphical plots and numeric reports of the estimated sample size (ESS). For this purpose Phase outputs were edited using Perl scripts designed by J.J. Gillespie, M.J., Yoder http://hymenoptera.tamu.edu/rna, slightly modified by the authors. Plots for the LnL of the stationary phase of each one of the models may be found in the additional file 3.

### Analysis using parsimony as optimizing criterion

Three different approaches were employed for analyzing data under the parsimony criterion. All employed the program POY 4.0 [58] since it also analyzes static alignments and morphological data using standard tree-searching algorithms, yielding results equivalent to programs like NONA and TNT. For analysis including only static alignment and morphology the results were checked using TNT [59]. Analyses were run at the 32-processor computer cluster held at Departamento de Zoologia da Universidade de São Paulo.

In the first, hereafter named 'traditional', analysis only the aligned nucleotides were included and gaps were scored as a fifth state. Heuristic searches were carried out using TNT employing 'New Technology search' (10000 random seeds, search including Ratchet and Tree-fusing, ran until the same strict consensus was hit ten times) and POY analysis (20 rounds of a POY script including the commands: build (320); perturb (iterations:10); swap (trees:1, annealing:(20, 2))).

In the second, a 'standard POY analysis' was performed employing direct optimization. Wheeler & Hayashi [9] and Giribet *et al. *[10] employed this approach for inferring chelicerate phylogeny. Direct optimization (DO) allows skipping of the intervening step of multiple sequence alignment by searching simultaneously for the best tree under an optimization criterion (in this case parsimony) and the nucleotide homology [60].

Similar to other automated programs for multiple alignments the POY final score is a function of a cost regime chosen *a priori*. In a standard direct optimization inference, as many cost regimes as possible are employed in independent runs and the one which minimizes incongruence among data partitions (different genes, genes × morphology, different gene regions etc.) is chosen [61]. These runs are also used to explore the behavior of data across the parametric space and, according to Giribet [62], is a way of evaluating node stability (*contra *[63]).

The SSU rRNA unaligned sequences were spliced into 12 blocks using conserved regions as a reference and, along with the LSU fragment, analyzed under direct optimization (DO) as implemented in the POY 4.0 program. For the sensitivity analysis the following cost regimes were tried (gap extention: tranvertion: transition ratios): 111, 121, 112, 211, 221, 411, 412, 421.

Search rounds using a POY script included alternate SPR and TBR after building 320 initial Wagner trees (build (320); swap (trees:1, annealing:(20,2))) and was repeated at least 20 times. Results from each one of these costs regimes were evaluated using congruence as a criterion. This was achieved using an ILD metrics (Incongruence Length Difference, [64]). For ILD calculations data partitions considered were the SSU and LSU rRNA sequences:

Jordal *et al. *[65] employed an interesting approach. They combined the secondary structure-based alignment to each of the RAA's in a POY analysis, analyzing each fragment in different data sets. This avoids violating positional homology where it may be inferred by secondary structure and explores the phylogenetic signal from regions where it is otherwise impossible.

A similar approach is employed in this study and compared with a standard DO analysis.

We have labeled this a 'constrained POY analysis'. For the RAAs, DO was employed with the same cost regimes employed in the standard analysis described above. For the pre-aligned regions, all transformations were equally weighted under static homology. We proceeded in this way because there is no objective justification for differential weighting in this case.

For the later analysis, the single strand RAA in the stems' tips were lumped with the REC. We proceeded in this way because the individual nucleotide homology cannot be accessed with confidence, due to possible exchange of nucleotides between REC and terminal loop RAA. The POY inputs for these analyses are presented in the additional file 4.

### Morphological and combined analysis

Morphological character statements were largely derived from the primary literature, authoritative reviews or direct observations. They are summarized in additional file 5 and any discrepancies between our own interpretations and previous hypothesis are discussed there. The full data matrix includes 178 characters and is hereafter named matrix A and presented in the additional file 6.

We were interested not only in knowing how combining the morphological matrix we gathered impacts the topology recovered by the combined analysis, but also in detecting any eventual limitations of direct optimization in maintaining the molecular characters' independence. For this purpose our results were compared with analysis combining the molecular data with a matrix produced applying Shultz's [22] character statements to the sampled taxa. This matrix - the most complete overview of arachnid morphological characters published thus far - is hereafter named matrix B - also presented in the additional file 6.

The combined analysis using the matrices A and B repeated the three analyses described above for the molecular data. The cost regimes tested were the same as the molecular analysis described above and the criterion of cost regime selection was also congruence as measured by ILD metrics. For a given cost regime other than 111, morphology was weighted according to the ratio of the molecular tree length obtained in this cost regime and that obtained in 111. It was made for trying to keep the morphological contribution to the final result approximately constant.

Results for the constrained analysis combined with matrix A is the preferred hypothesis. For this, after completing the sensitivity analysis, we used all resulting trees from all parameters for tree fusing [66]; a technique designed for avoiding heuristic problems [67].

It is important to bear in mind that re-sampling measures of support in the DO context is not directly comparable to that in a static homology context, since the first sampled entities are sequence fragments and not individual nucleotides. Thus, for evaluating branch support, Bremer supports were considered more informative and were calculated for the constrained analysis combined with matrix A.

## Results

### Data characteristics and rRNA secondary structures

**Figure 2:**
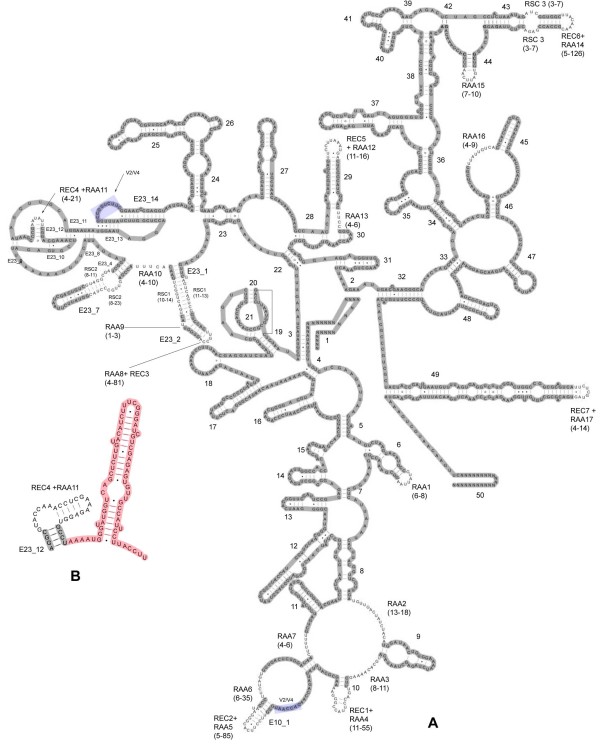
**SSU secondary structure of exemplar species**. Regions alignable across the dataset are shaded gray. A- *Rostrozetes ovulum *SSU secondary structure. Regions of ambiguous alignment have their length variation indicated in parenthesis. The Helix V2/V4, a tertiary interaction is shaded blue. B- Detail from the V4 region of *Tenuipalpus heveae*, including the Helix E23_12. The red shaded area is excluded from the constrained POY analysis since it lacks detectable homology with other Chelicerata.

The SSU rRNA sequences in the 91 species in this study ranged from 1713-2154 nucleotides from helices 5 to 49; the longest sequences being observed in Tetranychoidea (*Tenuipalpus hevae*). The length variable regions are plotted against the secondary structure inferred for *Rostrozetes ovulum *(Acariformes, Oribatida, Fig. 2A) which is consistent with the general model for eukaryotes [40]. Nucleotides included in the aligned matrix are shaded in gray. Combined, they comprise 1581 positions, 747 being conserved, and among the remaining 813 variable positions 568 are parsimony informative.

When combined, all regions of ambiguous alignment (RAAs, RECs and RSCs) ranged from 171-610 nts. If Tetranychoidea are excluded we observe a range from 171-263. Despite their length, Tetranychoidea SSU rRNA molecules do not present major changes in the secondary structure as the length variations are related to the hyperextension of RECs. An exception is a putative new helix in the V4 region (Fig. 2B, depicted in red). This helix, which comprises 49-66 nts, should be considered exclusive to Tetranychoidea and hence is excluded from the analysis which considered the secondary structure information.

**Figure 3:**
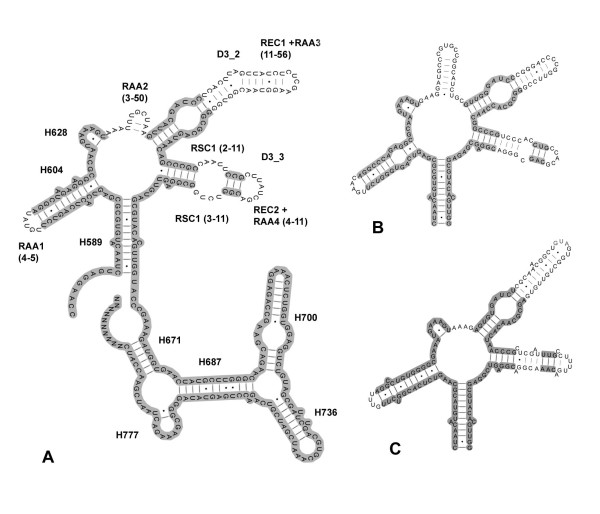
**LSU D3 region secondary structure of exemplar species**. A- Model for the secondary structure of the Oribatida mite *Rostrozetes ovulum*. Gray-shaded areas refer to alignable regions. B- Region D3 of the Scorpion *Androctonus australis*, note presence of helix D3_1. Blue-shaded region refers to the D3_1 helix present in most Chelicerata, number of nts associated with RAA2a and RAA2b refers to them alone. C- Region D3 of the Prostigmata *Halacarus *sp., note that similarly to *R. ovulum *in that it lacks a D3_1 helix.

The LSU rRNA fragment ranged from 284-350 nts. The largest inside-order variation was obtained among spiders: 284 nts for the Pholcidae species and 349 for *Atypoides riversi*. The secondary structure inferred is consistent with that proposed for eukaryotes by Schnare *et al. *[45]. A notable secondary structure variation is the absence of the D3_1 helix in Pholcidae, Tetragnathidae, and all Acariformes. Although some Acariformes species possess potential base pairing in the corresponding region (see e.g. *R. ovulum *in Fig. 3A), they do not exhibit covariation for postulating a helix in this region for the order. This region includes 3-34 nts in Acariformes; five in the Pholcidae species; four in the Tetragnathidae species; and 17-50 nts in species which present a D3_1 helix.

The replacement of one secondary structure for another may be regarded as alternative states of a qualitative character. Thus a morphology-like character was added to the analysis, scored as 1 for taxa in which the D3_1 helix is absent and 0 for those in which it is present. Furthermore, individual nucleotides comprised in these different structures are not directly comparable and were set into different files when integrated into the POY constrained analysis.

Finally, when the D3_1 helix is taken in isolation, the covariation along the basal portion of the helix allows inference of the nucleotide homology for the blue-shaded nts in Fig. 3B. Hence, the region was divided in three smaller sequences; one treated as pre-aligned and two under direct optimization.

The aligned data set derived from the LSU sequence contained 256 positions, of which 95 were conserved and 125 parsimony informative.

The combined 18 S rRNA plus 28 S rRNA pre-aligned data set has a mean base composition across the entire matrix as follows: U = 23.1; C = 22.3; A = 26.4; and G = 28.2. When only loops are taken into account the composition is U = 22.7; C = 17.1; A = 38.3; and G = 21.9, while for stems it is U = 23.5; C = 26.6; A = 16.7; and G = 33.2. This compositional bias toward adenosines in loops was already observed in several datasets, and is explained by the high percentage of unpaired adenosine nucleotides in several structural motifs [68].

### Molecular analysis based on static molecular homology

**Figure 4:**
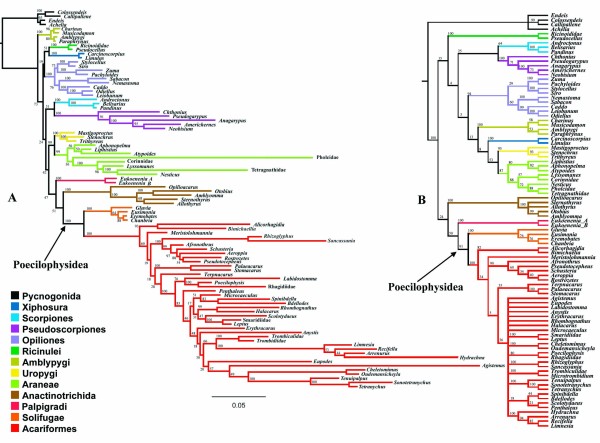
**Molecular analyses including only pre-aligned regions**. A- Bayesian phylogram. Pairing and non-pairing regions were modeled separately. A seven-state model (7A) was implemented for the paring regions and a GTR + I + G was used for the non-pairing regions. Numbers indicate posterior probabilities. B- Strict consensus of the 28 most parsimonious trees of molecular data under static homology as established using secondary structure as a guide (MPT, Length = 4982; CI = 0.338; RI = 0.600).

Bayesian analyses, considering the stems and loops as separate partitions, improved the lnL over the uniform GTR +I +G model (lnL harmonic means of -25881.12 and -26073.89, respectively, B_10 _= 192.76). But the Bayes factors clearly favor the mixed models that apply base pairing models for stems: GTR +I +G\16A and GTR +I +G\7A, with an almost identical harmonic mean for lnL (-21674.70 and -21675.42). All Bayesisan analyses, however, presented similar topologies concerning well-supported taxa. The phylogram presented here was recovered by the GTR +I +G\7A model (Fig. 4A).

The traditional parsimony based analysis, both employing POY and TNT reported 28 trees with 4982 steps (CI = 0,338; RI= 0,600) when considering gaps as a fifth state. Jackknife supports were calculated as presented in Fig. 4B.

The Bayesian and parsimony analysis recovered almost all interordinal relationships with weak support (Jackknife > 50%, Posterior Probabilities > 75%) except for Uropygi (Thelyphonida + Schizomida) (J. = 95%, P. P = 100%) and Solifugae + Acariformes (J. = 91%, P. P. = 100%). All traditional orders except Acari, which resolve split into Acariformes and Parasitiformes, were recovered with high support; as well as Euchelicerata, but interestingly not Arachnida. Neither Labellata (Amblypygi + Araneae) nor Pedipalpi (Amblypygi + Uropygi) is recovered but (Uropygi + Araneae) is recovered with moderate support in the parsimony analysis (J. = 53%) and high support in Bayesian analysis (P. P = 99%).

Concerning Acariformes intraordinal relationships, it is noteworthy that Bayesian analysis recovers some traditionally held taxa with high support which are not recovered, or recovered with low support, in the parsimony analysis. This is the case for the families Bdellidae (J. = 44%, P. P = 83%), Halacaridae (P. P = 99%), Astigmata + Oribatida Brachypylina (P. P = 100%), and Prostigmata (P. P = 100%).

On other hand, parsimony analysis recovers with high support a Tetragnathidae + Pholcidae clade among spiders (J. = 88%) while Bayesian analysis places Pholcidae basal among Araneomorpha with Tetragnathidae as sister to *Nesticus*; thus supporting Orbiculariae (P. P = 100%) and Entelegynae (P. P = 100%). The Bayesian analysis estimated long branches leading to both Pholcidae and Tetragnathidae and their association in the parsimony analysis is regarded here as a long branch attraction artifact. Concerning spiders, however, Bayesian analyses oddly do not support Mygalomorpha monophyly.

### Morphological analyses

**Figure 5:**
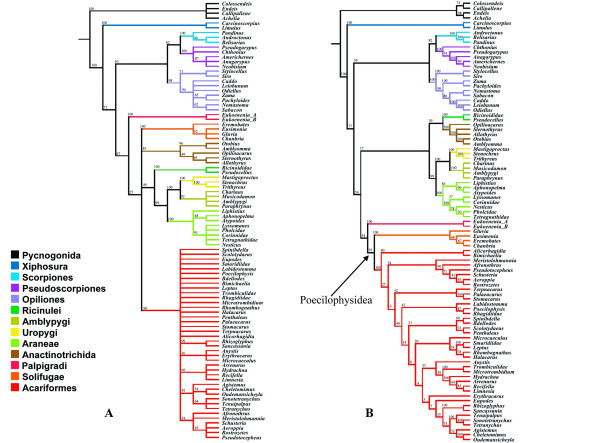
**Combined analyses of aligned molecular data and matrix A**. **A- **Strict consensus of the 233 MPT recovered from the analysis based on the morphological matrix A, assembled during the present study (Length= 385; CI= 0.564; RI = 0.922) **B- **Strict consensus of the 8 MPT recovered by the analysis based on the matrix A and the aligned molecular data (length = 5411; CI = 0.352; RI = 0.663).

**Figure 6:**
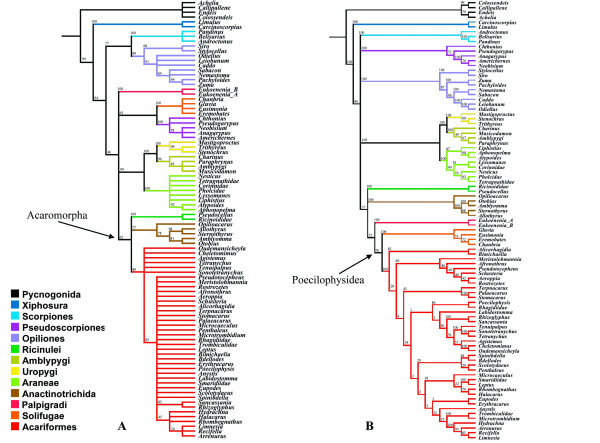
**Combined analyses of aligned molecular data and matrix A**. **A**- Strict consensus of the 1482 MPT recovered from the analysis based on the morphological matrix obtained from Shultz's (2007) study (Length= 420; CI= 0.548; RI = 0.913) **B**- Strict consensus of the 4 MPT recovered by the analysis based on the morphological matrix B and the aligned molecular data (length = 5447; CI = 0.352; RI = 0.661).

As stated in the methods section, two different morphological data matrices were assembled for testing the impact of morphological data sets on the combined analysis. The trees recovered from the analysis of both matrix A and B are similar in supporting Amblypygi as sister group of Uropygi (Pedipalpi), Tetrapulmonata, and Arachnida (Figs. 5A and 6A, respectively).

Analysis of matrix A (characters recognised here) recovered neither Acaromorpha *sensu Shultz *nor Haplocnemata. Palpigradi was recovered in a basal position relative to a polytomy composed of (Tetrapulmonata + Ricinulei), Parasitiformes, Solifugae, and Acariformes (Fig. 5A). Pseudoscorpiones resolve here as the sister group of Scorpiones instead of Solifugae, both associated with Opiliones.

Analysis of matrix B (Shultz's data) led to results similar to the original Shultz [22] analysis. It recovered Scorpiones as sister group of Opiliones, and a polytomy composed of Palpigradi, Tetrapulmonata, Acaromorpha *sensu *Shultz (Ricinulei, Acariformes and Parasitiformes), and Haplocnemata (Pseudoscorpiones + Solifugae) (Fig. 6A).

### Combined morphological and molecular analysis under static homology

In the combined analysis under static homology (Figs. 5B, 6B), Solifugae was recovered as the sister group of Acariformes mites irrespective of the morphological matrix employed. Tetrapulmonata was also recovered in both analyses. Neither matrix A nor B led to a well supported position for Ricinulei plus Parasitiformes mites, but the combined analysis employing matrix A recovered a taxon composed of (Opiliones (Pseudoscorpines Scorpiones) (J. = 59%). These three orders appear in the topology recovered using matrix B in a polytomy. Palpigradi is recovered as sister group of (Solifugae Acariformes) in both analyses, with Jackknife supports of 54% and 37% respectively.

### Molecular analysis integrating Regions of Ambiguous Alignment

**Figure 7:**
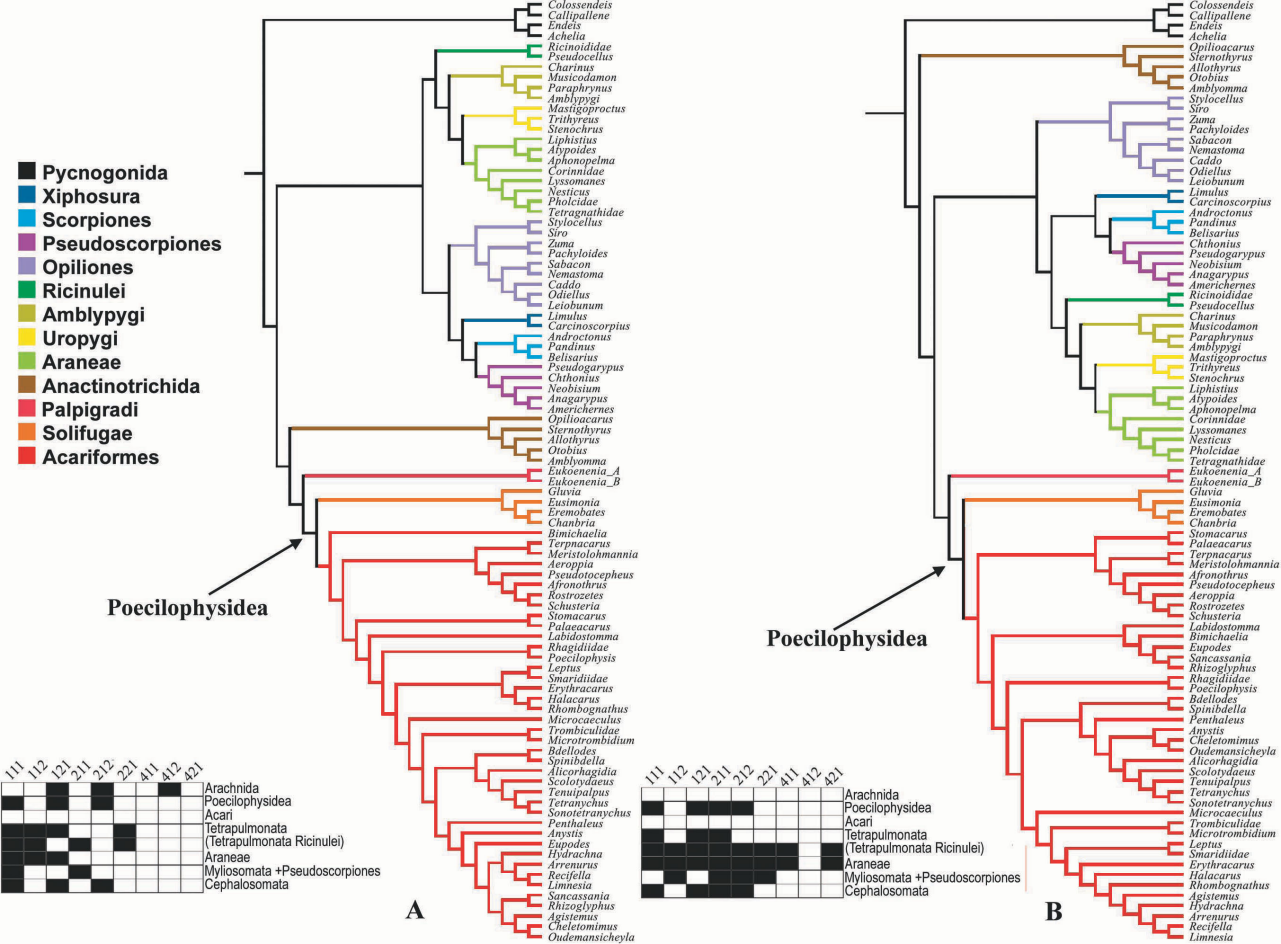
**Molecular analyses including RAA**. **A- **Single MPT, with 9998 steps of length (CI= 0.434; RI = 0.676) recovered from the standard POY analysis based on molecular data only and respective plotting of the sensitivity analyses. **B- **Single MPT, 10095 (CI= 0.412; RI = 0.638) steps long recovered from the constrained POY analysis of molecular data and respective plotting of the sensitivity analyses.

The first noteworthy difference between the standard and secondary structure constrained POY analysis was the computational time required for similar searching strategies, with the former taking approximately 8.7 times longer than the later using the same number of processors. The standard POY analysis recovered a single more parsimonious tree with a length of 9998 steps, CI= 0.434, RI = 0.676 (Fig. 7A). The cost regime to minimize incongruence was that with all changes equally weighted (ILD metrics summarized in Additional file 7). The constrained analysis yielded a single MPT, 10095 steps long, with CI= 0.412, RI = 0.638, incongruence leading to the choice of the same 111 cost regime (Fig. 7B).

The optimal topologies are similar with respect to most interordinal relationships. They both present the clades (Palpigradi (Solifugae Acariformes)), (Scorpions Pseudoscorpions), and (Ricinulei (Amblypygi (Araneae (Thelyphonida Schizomida)))).

All traditional orders, except Acari, are recovered in both analyses at the optimal cost regime. However, only the cost regimes 111, 112, 121, and 221 recovered Araneae in the standard analysis. The order is not supported under the cost regime 412 in the constrained analysis only. Parasitiformes is oddly recovered as a basal offshoot in both analyses.

### Unconstrained combined analyses

**Figure 8:**
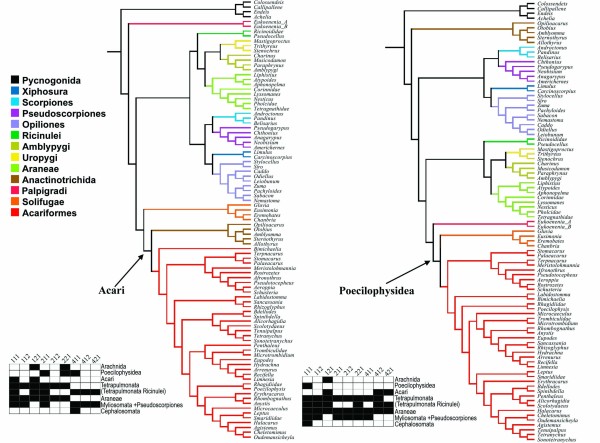
**Combined analyses including RAA and morphological matrix B**. **A- **Strict consensus of the two MPT with 10491 steps of length (CI = 0.434; RI = 0.685) recovered from the standard POY analysis of molecular data combined with the Shultz-based matrix and respective plotting of the sensitivity analyses. **B- **Single MPT, 10587 steps long (CI = 0.411; RI = 0.659) recovered from the constrained POY analysis of molecular data combined with the same Shultz-based matrix and respective plotting of the sensitivity analyses.

**Figure 9:**
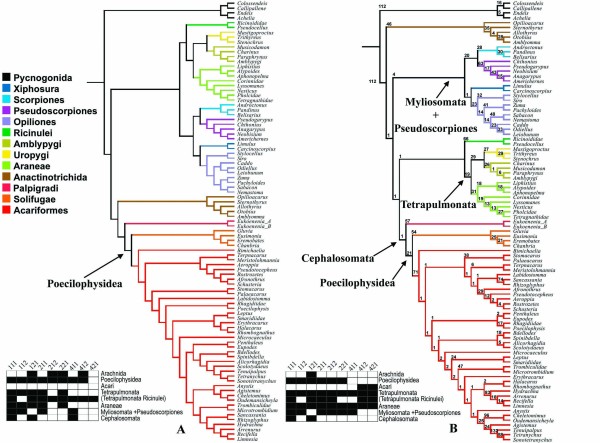
**Combined analyses including RAA and morphological matrix A**. **A- **Strict consensus of the two MPT with 10451 steps of length (CI= 0.432; RI= 0.694) recovered from the standard POY analysis of molecular data combined with the morphological matrix assembled in the present study and respective plotting of the sensitivity analyses. **B- **Single MPT, 10548 steps long (CI = 0.412; RI = 0.666) recovered from the constrained POY analysis molecular data combined with the same morphological matrix assembled in the present study and respective plotting of the sensitivity analyses. Values associated with the branches represent Bremer support.

The unconstrained combined analyses are remarkable for presenting ILD values almost identical for the costs regimes 111 and 121. The former cost regime was chosen arbitrarily for presenting the topologies in Figs. 8A and 9A. Results differ between the cost regimes no less because the later presents Arachnida as a monophyletic group. The ILD metrics for all analyses are summarized in Additional file 7.

Combined unconstrained analyses of the molecular data and matrix A yielded a single MPT with 10451 steps, CI= 0.432, RI= 0.694 (Fig. 8A). When combined with matrix B, the unconstrained analysis recovered a single MPT, with 10491 steps, CI = 0.434; and RI = 0.685 (Fig. 9A).

The former yielded Solifugae as sister group of Acariformes. The latter did not recover the (Palpigradi (Solifugae Acariformes)) clade found in the purely molecular analyses and remaining combined analyses. Instead, Acari was recovered as monophyletic and associated with Solifugae. Furthermore, Palpigradi was recovered as basal, i.e. as sister group of all other Euchelicerata orders.

### Constrained combined analyses

The ILD metrics unambiguously point to the cost regime 111 as optimal for both matrices A and B. While the unconstrained analyses yielded considerable change in optimal topology with respect to the morphological matrix with which it was combined, the secondary structure constrained analyses resulted in the same interordinal scheme of relationships; albeit with different frequencies along the sensitivity analyses. The analysis combined with Shultz's matrix B resulted in a single MPT with 10587 steps in length (CI = 0.411; RI = 0.659, Fig. 8B), the analysis combined with matrix A yielded to a single tree with 10548 steps in length (CI = 0.412; RI = 0.666; Fig. 9B). The resulting topology is discussed in further detail below and the respective Bremer support values are displayed for its branches in Fig. 9B.

## Discussion

### Methodological remarks

One of the most exciting aspects of phylogenetic studies is that methodological concerns should, more often than not, be addressed simultaneously with the phylogenetic inference. Except for bizarre exceptions, such as short term viral evolution, true phylogenies are unknown. The alternative employment of simulated data or well-supported phylogenies must not preclude the researcher thinking about their own practice.

Shultz [22] brought our attention to the fact that "There is a tendency to portray arachnid ordinal phylogeny as more poorly resolved and contentious than is actually the case". If only the morphological and combined analyses are taken in consideration, this is certainly the case, although the results obtained by purely molecular analyses depart considerably from this convergence.

A possible explanation for this apparent contradiction is the way that molecular homology was established in those combined analyses that preceded us. Direct optimization was already criticized on the grounds of its possible inaccuracy in recovering nucleotide homology when compared with more traditional algorithms such as Clustal W [69] (*contra *[70]) and secondary structure alignment [71]. Our combined analyses results, however, may be better explained by another drawback, the lack of independence among data partition, which we here discuss further.

As in other comparative sciences, systematics is based on the concept of homology. The formulation of a hypothesis of character homology is a two step process: (a) from observations and knowledge external to the analysis researchers propose characters and their states, after which (b) several independently formulated characters are assembled into a matrix and subjected to cladistic analysis. Only after the phylogenetic analysis is it possible to distinguish between similarity due to convergence and shared ancestry, by using character conflict or conformation to the optimal topology [72]. This double nature of homology is further discussed by Assis & Brigandt [73].

In molecular systematics, primary homology assessment is accomplished by multiple sequence alignment, which is crucial for ribosomal genes that may vary in length by hundreds of nucleotides. Multiple alignments have been considered a computational rather than a biological issue and much more effort has been employed in improving algorithms for matching individual nucleotides under a similarity criteria than to address what evidence must be pursued for aligning nucleotides that share evolutionary descent (see Morrison [74] for a revision).

Length variation yields considerable phylogenetic signal [75], but also produces uncertainty in nucleotide homology. Usually, regions of ambiguous alignment (RAA) are detected by alignment inspection and then discarded. Along with these discarded regions, any phylogenetic signal they might have contained is obviously lost. To avoid such 'wastage' several methods were proposed for incorporating RAA into the phylogenetic analysis [76].

Direct optimization circumvents it by inferring phylogeny and homology simultaneously, in a 'one step' approach. Wheeler & Giribet [77] made clear their judgment on multiple alignments and considered it a non-scientific procedure. In their opinion molecular data must be analyzed as they are obtained from sequencing. In fact, proponents of POY consider multiple alignments to be more than useless; even deleterious to the analysis:

"In the same way that each cladogram has a (potentially) unique set of optimal character origins, each cladogram may have a unique set of optimal correspondences among observed features. Unless these correspondences are unrestricted and allowed to be optimized together with transformations, biased and conditional results may be obtained. Such bias may come from assumptions of the investigator and his or her notions of appropriateness of comparison, and conditioned on the hypotheses most in agreement with preconceived correspondences of 'primary' homology" (Wheeler *et al*.[[78], p. 11]).

Wheeler and colleagues advocate instead an approach to systematics classified as 'instrumentalist' by Rieppel [79], which embraces a specific stand point on the nature of evidence in phylogenetic inference. For these authors, evidence must enter directly into the analysis without being filtered by the (ever misleading?) researcher subjectivity, or constrained by considerations external to the phylogenetic analysis itself.

The reasoning elaborated by Simmons [80] led us to reject this perspective and is especially relevant for discussions concerning Shultz's [22] perception of convergence of results and the analyses presented here. Simmons [80] argued that the primary homology assessment step is an insurmountable requisite for phylogenetic analyses because it is a guarantee for maintaining character independence.

POY uses parsimony for optimizing nucleotide homology and congruence for parametric regime choice. Both parsimony and congruence require that characters are independent within and among data sets. If a character state lies on the state of another character, a single evolutionary event will be over-weighted. If character states in a data set lie on the character states in another dataset it is likely that the combined analysis will be biased toward a higher congruence among these datasets.

Character dependence may be the product of a shared causal mechanism, like sharing the same selective pressures. However, Simmons [80] argued that when combining unaligned sequence with static homology data (i.e. pre-aligned sequences or morphology) using POY, the final result will be biased toward the signal provided by the data under static homology. The cause of dependence in this case is not related to the underlying biological nature of the character, but rather by the way direct optimization works when establishing character homology in its 'one step' approach to phylogenies. The core of his criticisms on direct optimization is that such influence is sufficient to blur the phylogenetic signal of the unaligned molecular data. He argued that while optimizing tree length, potential conflict among molecules being analyzed under DO and characters analyzed under static homology may be erased by the algorithm moving unaligned nucleotides around. Rieppel [79] presented similar arguments and referred it as "the fluidity of character statements".

Usually, morphological data has a limited effect when combined with molecular data (see e.g. [81], for its impact on resolution and support). Morphological influence is arguably limited by the relatively small number of characters. However, during a standard POY analysis morphological data also contributes to the final topology by: (a) its effect on establishing the molecular homology under a given cost regime and (b) on the choice of the cost regimes, since this decision is taken on grounds of minimizing incongruence.

**Figure 10:**
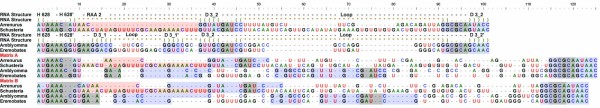
**Sensitivity of homology assessment to the morphological matrices**. Sequences from the D3 region ranging from helix H628 to D3_2 of two Acariformes mites (*Arrenurus *and *Schusteria*), Anactinotrichda (*Amblyoma*) and Solifugae (*Eremobates*). A- Partially aligned sequences, with regions aligned according to the secondary structure shaded blue. Parenthesis indicates pairing sites, dots unpaired sites and (*) indicates regions considered unaligned. Note that D3_1 is absent in Acariformes. B- Implied alignment bound to the best trees of the unconstrained analysis of molecular data combined with matrix A, under cost regime 1:1:1. C- Implied alignment, under the same cost regime, analysis combined with the matrix B.

This suggests that lack of independence could explain the disparity between the position and monophyly of mites when combined analysis results are compared with exclusively molecular analysis, as per Wheeler & Hayashi [9] and, mainly due to their improved sampling effort, in Giribet *et al *[10] and in our own analysis employing unconstrained direct optimization. It is exemplified in Fig. 10, where implied POY alignments correspondent to the trees recovered in the analyses combined with matrix A and B are compared, showing that molecular homology statements are diverse between the two analyses.

Although phylogenetic inference cannot be ascribed to a falsification context [82] (for an alternative standpoint see [83]), researchers are interested in at least verifying how plausible current hypotheses of relationships are. The 'epistemological interdependence' created by direct optimization makes this 'test' less rigorous and may preclude the discovery of potential homoplasy. For example, convergence of the gnathosoma between Acariformes and Parasitiformes is recovered in the constrained analyses of the present study, but not in the unconstrained one applying the Shultz [22] based morphological matrix.

POY may share drawbacks with other automated multiple alignment programs. Hickson *et al. *[84] showed that any multiple alignment method which works at the individual nucleotide level is incapable of recovering homology relationships inferred from secondary structures in rRNA sequences, and this is clear in Fig. 10. The results of the present study have reinforced the potential of secondary structures to help formulate more accurate hypothesis of homology. This is indicated by the similar values of incongruence in the constrained analysis, a surprising result since POY seeks to minimize incongruence. Two clear examples of how secondary structure may be employed for data partition may explain this. Other interesting examples of how use secondary structure information in an analysis may be found in Swain & Taylor [85].

In the first example, an extra helix, absent in all other Chelicerata could be determined in the Tetranychoidea species (Fig. 2B). The emergence of these structures has been documented ever since initial studies on secondary structures of ribosomal genes were carried out, e.g. [86]. The nucleotides contained in this new structure are arguably non-homologous to any others found among the remaining species, yet they are tentatively aligned automatically with nucleotides in other sequences using any of the cost based alignment algorithms, including POY.

In the second example, with more fundamental consequences for the present analyses, there are length convergences among some taxa. In Tetragnathidae and Pholcidae (both spiders), and Acariformes mites, which have convergently lost the D3_1 helix in the LSU fragment studied. We must also consider *Atypoides riversi *and Ricinulei which have large insertions in different, but contiguous, helixes (the LSU D3_2 and D3_3). These length convergences are easily amenable to homology establishment using secondary structure as a guide. In the spider-acariform case, the presence/absence of the D3_1 helix is considered to be alternative states of a same character. In the second case, *Atypoides *and Ricinulei had their insertions set in different files. Otherwise, this situation led to spiders being recovered in just four cost regimes (111, 112, 121, 221), and oddly (Pholcidae, Tetragnathidae, Acariformes) in 411, 412, and 421 and (*Atypoides *Ricinulei) in 211 and 212.

Integrating the regions of ambiguous alignment - ambiguous relative to the criteria adopted here for homology establishment - brings to the present analyses phylogenetic information which both supported Tetrapulmonata and placed Ricinulei as it sister group; two clades which have been suggested based on other lines of evidence (see below). It leads us to support the inclusion of these regions into phylogenetic analysis as argued by Giribet & Wheeler [75], but with a note of caution when interpreting the results.

Any alignment is an inference, since gaps are not observed from sequencing results. Therefore, alignment includes a variable degree of uncertainty. In a standard analysis, where only regions regarded as unambiguously aligned are considered, this uncertainty is comfortably ignored by those who perform the analyses.

It is harder to do so considering the regions of ambiguous alignment. POY provides point estimation on the optimal alignment for these regions given a cost regime, but is uninformative on the impact of homology inference uncertainty on node support.

Furthermore, parameter choice is made among a limited set of cost regimes applied uniformly to the sequence and not estimated by the sequences properties. Redelings & Suchard [87] proposed an algorithm for simultaneously estimating alignment and phylogeny, taking into account alignment uncertainty that circumvents this problem in a Bayesian frame. It is expected that such developments will allow further integration of RAA's phylogenetic information while at the same time considering the uncertainty in support estimation. Currently, however, the implementation of this method is practical only for smaller data sets.

Given the considerations above, the hypothesis that combines (a) a better knowledge of rRNA properties when establishing homology, (b) keeps the among-partition independence and (c) encompasses the information from the RAAs, is that recovered under the constrained analysis combined with matrix A (Fig. 9B). This hypothesis will be the basis for the following discussion.

### Phylogenetic position of Acariformes

Acaromorpha *sensu *Shultz is supported neither by the morphological nor the molecular data presented here. In fact, both converge in placing Ricinulei as the sister group of Tetrapulmonata (i.e. spiders and their closest relatives), although with low Bremer support in the final analysis (Fig. 9B). Among the putative synapormorphies for (Ricinulei + Tetrapulmonata) we have the presence of a tritosternum, a feature also shared by several Parasitiformes mites (Ch. 12), a cheliceral apotele articulated dorsally (Ch. 28), loss of the proximal segment of the chelicerae, convergent with Acariformes mites, Solifugae and Pseudoscorpiones (Ch. 27), coiled sperm cells, shared with Pseudoscorpiones (Ch.162) and the presence of a machete of microtubules associated with the spermatid nucleus (Ch. 167). The association of Ricinulei with Tetrapulmonata is further supported by fossil data. Ricinuleids share several putative apomorphies with Trigonotarbida; a whereby (Trigonotarbida + Tetrapulmonata) together form the Pantetrapulmonata *sensu *Shultz [22]. As in the ground plan of Tetrapulmonata, trigonotarbids also have two pairs of book lungs in opsithosomal segments 2 and 3 respectively [88], as well as the typical tetrapulmonate 'clasp-knife' chelicerae. Explicit morphological characters supporting (Ricinulei + Trigonotarbida) include palpal chelae where the apotele opposes the tarsus (Ch. 41), presence of a locking mechanism between the prosoma and opisthosoma (Ch. 68), longitudinally divided opisthosomal sclerites (Ch. 69), and the presence of a diplosegment formed by the fusion of opisthosomal tergites 2 and 3 (Ch. 70) [18,19].

As discussed above, a monophyletic Acari could not be recovered in those cost regimes that minimized incongruence, except when combining the unconstrained data with matrix B. The position of Parasitiformes could not be further addressed here. In fact, we should regard this as a 'wild card' group. The basal position recovered from the present data is arguably an artifact and has no morphological support. An alternative hypothesis would be a sister group relationship between Acariformes and Ricinulei. This is the so-called Cryptognomae hypothesis, introduced by Hammen [13], with their putative sister group in his scheme being Trigonotarbida. Another possibility would be to treat anactinotrichid mites as a basal offshoot from the lineage leading to the Tetrapulmonata + Ricinulei group. The latter model would receive support from the presence of a tritosternum (Ch. 12) and the way coxal glands fluids reach the pre-oral chamber (Ch. 115). Finally, numerous molecular analysis have recovered Parasitiformes as the sister group of Pseudoscorpiones [10,11,23,24]. Characters such as the fusion of the labrum to the epistome, and a ventrally placed cheliceral apotele support this hypothesis (Ch. 13, 28), although neither of these traits are exclusive to them. It is noteworthy that all these mentioned analyses have not included basal Pseudoscorpiones, restricting themselves to members of the more derived Iocheirata.

### Solifuges and acariform mites

The most significant result from the present combined study is a strong signal for a sister group relationship between Solifugae and Acariformes. Interestingly, the same result was obtained in the recent molecular tree of Dabert et al. [24] with similarly high support values; albeit using a slightly different set of genes (specifically we used D3 LSU rather than COI).

Dabert et al. [24] further discussed morphological support for this clade, mentioning similarities in the position of the tracheal openings of solifuges and at least the prostigmatid mites, or the potentially homologous lateral organs of Solifugae and the Claparède organ of Acariformes. From our character set we recognize the following putative apomorphies: a narrowing of the sternal region, the fusion of the labrum to the epistome, and a ventrally placed cheliceral apotele (Ch. 7, 13, 28). Some of these character states are recovered as convergent with, respectively, Pseudoscorpiones (Ch 7), and/or Pseudoscorpiones and Parasitiformes mites (Ch. 13, 28). Exclusive to Solifugae and Acariformes are the putative synapomorphies of loss of the nuclear envelope during spermiogeneis (Ch.158, although a somewhat similar condition may occur in some Xiphosura), and the presence of a specific structure of the testis (Ch. 168).

This apomorphic histology of the testis is the most striking feature uniting solifuges and acariform mites [20,89]. This feature was overlooked by the Shultz [22] study, in which he accused (p. 236) Alberti & Peretti, and other workers, of trying to "support specific (target) clades". Despite such criticisms it is interesting to note that the explicit character of testis morphology which Alberti & Peretti formally proposed in support of solifuges and acariform mites was conspicuously absent from Shultz's own morphological matrix. One can also target clades by excluding data *a priori*.

Considering the testis character in detail, in Parasitiformes mites, for example, spermatogenesis progresses in a roughly anterior-posterior direction along the testis, and spermatogonia are observed in adults. Secretory cells are lacking in the testis. In Acariformes, by contrast, no spermatogonia are observed in adults and meiosis putatively occurs only in juveniles. Furthermore, the testes have a dorso-ventral orientation with a dorsal germinative region delivering sperm cells to the lumen, apparently by germinative epithelia fragmentation, while the ventral secretory specialized epithelia deliver a sperm-accompanying secretion through a more or less developed brush border. This dorso-ventral orientation is also clear in spermatid development with mature sperm cells restricted to the ventral portion of the germinative region. Exactly the same condition is observed in Solifugae, except that here only mature sperm cells are documented in adults.

As noted by Dabert et al. [24], a possible relationship between solifuges and acariform mites also has historical precedence [90]. Cambridge [91] described a rhagidiid mite as a new arachnid order which he named Poecilophysidea; considering it a mite-like animal, but with solifuge-like characters. Banks [[92], pp. 21-22] later claimed with reference to *Rhagidia*: "Its structure is in many ways very similar to that of certain solpugida and suggested to Thorell its generic name, which is a diminutive of *Rhax*, a genus of Solpugida. It is probable that, it is the most primitive of all existing mites, and points to a close relationship of the Acarina to the Solpugida." Rhagidiids do look, at least superficially, rather like tiny solifuges. While most cladistic work on mites - including the present study - has not recovered Rhagidiidae in a particularly basal position among the Acariformes, Dabert et al. [24] mentioned that this family was recovered either basal within the Eupodides clade or even basal among Trombidiformes; at least under some parameters of analysis. Given the new phylogenetic hypothesis linking solifuges and mites, further tests of the position of rhagidiids would be welcome.

Alternatively, Grandjean [93,94] drew comparisons between solifuges and another group of acarifom mites; the probably basal oribatid lineage Palaeacariformes. Grandjean highlighted similarities such as a dorsal sclerite (or propeltidium) associated with the first four pairs of appendages (our Ch. 1), the projecting mouthparts (Ch 13) and the Claparède/lateral organ (but see our Ch 175). As noted by Dunlop & Alberti [6], high-quality morphological studies incorporating both mite and non-mite arachnids are largely lacking and we hope that the results of the present analysis will encourage further comparative research of this nature. In this study, we choose to name the putative clade encompassing Solifugae and Acariformes as Poecilophysidea, in recognition of Cambridge's the early acknowledgement of the similarity between the two orders.

### The palpigrade problem

Palpigradi is one of the least known of the extant arachnid orders. Certain characters, such as the morphology of the endosternite, have led some authors to consider them as basal Arachnida [95]. Alternatively, palpigrades have previously been considered closely related to Acariformes mites [23,96] or Tetrapulmonata [16]; while Shultz [22] recovered them unresolved with respect to his other major arachnid lineages. The position of Palpigradi which is most stable, although with weak support, in the present analysis is as sister group of the Solifugae + Acariformes clade: a hypothesis we will name hereafter as "Cephalosomata".

The name Cephalosomata highlights the absence of a unitary carapace covering the first six appendage-bearing segments. Instead, as in Pycnogonida and Schizomida the group Palpigradi, Solifugae and Acariformes (due its sejugal furrow) present the four anterior appendage-bearing segments covered by a shield variously named the cephalosoma (which technically refers to the body region) or the propetildium (the dorsal shield itself) [97].

Significantly, Cephalosomata is only recovered when molecular data is brought into the analyses, but shares, besides the cephalosoma/propetidium (Ch. 1), the absence of a sperm cell flagellum (Ch. 161, convergently lost in Parasitiformes and Phalangida harvestmen and scored based on *Prokoenenia*, since the genera *Eukoenenia *has not been studied with respect to this feature); presence of a secretory region on the coxal glands (Ch. 115) and the putative number of body segments (Ch. 66); both character states modified or lost in Acariformes. These characters are admittedly highly homoplastic and possibly under-studied, yielding only weak morphological support for this clade. The constrained analysis combined with matrix A also recovered Cephalosomata, although with a low Bremer support (Fig. 9B).

Despite this, we suggest that some aspects of Cephalosomata morphology may yield novel data for the group. Solifugae is unique among Arachnida for displaying the most complete set of embryological opisthosomal appendage buds, comprising transitory appendages from the first to the tenth opisthosomal segments [98]. Most of them degenerate quickly, but tracheal stigmata develop behind the 3^rd ^to 5^th ^segments. These same segments develop the putative respiratory lung sacs (or 'ventral sacs') in some Palpigradi, but since embryology is largely unknown for this group it remains equivocal as to whether these sacs in palpigrades are appendage derivatives too. 'Ventral sacs' are often treated as homologous to similar structures seen in Amblypygi [99], but they were regarded by Hammen [96] as homologous to the genital acetabula in Acariformes due their similar post-embryonic development.

The appendicular nature of the genital acetabula is not demonstrable from traditional embryological observations of Acariformes, since appendage buds in the appropriate position have never been recorded for this group. Yet the acetabula share the same fine structure and function as the epimeral pores or Claparède organs [100,101], which are demonstrably exopod derivatives among the mites. In summary, Palpigradi 'lung sacs', Solifugae spiracles and Acariformes genital acetabula may be vestigial expressions of the same appendages on the same body segments. Moreover, we could speculate that the trilobated genital opening in both Palpigradi and Acariformes are homologous structures; as did Hammen [96].

### Arachnida and future prospects

Finally, molecular data do not support a monophyletic Arachnida. In the optimal hypothesis, Xiphosura are recovered in a group including Scorpiones, Pseudoscorpiones and Opiliones. We should note that the last three orders are recovered together mainly thanks to muscular appendicular characters [15] in our morphological analysis (Fig. 5A). This clade recalls Dromopoda, *sensu *Shultz [16], but here excluding Solifugae and including Xiphosura, or Hammen's 'Myliosomata' (here including Pseudoscorpiones); see Hammen [14] for a discussion of this group defined on 'coxisternal' feeding.

Challenges to arachnid monophyly have usually faced much criticism (see especially Shultz [16,22,102]) and a problem already experienced in similar analyses including extant material is the fact that many putative chelicerate plesiomorphies are unrecognizable among Pycnogonida. This has led to analyses optimizing these characters as autapomorphic for Xiphosura. We may list in this context the presence of a carapace pleural margin (Ch. 3), a cephalic doublure (Ch. 9), a posteroventraly directed mouth (Ch. 11), a well developed post-anal telson (Ch. 23), presence of gnathobases (Ch. 47) and presence of endosternal suspensors of opisthosomal somites I and II (Ch. 125). All these characters are not clearly applicable to, or else wholly absent from, Pycnogonida. However they are (in part) evident in potential chelicerate outgroups ('trilobitomorphs', 'great appendage' arthropods) among the early Paleozoic arthropods [103]. Integration of paleontological data will be crucial for solving the problem of character polarity within Chelicerata - and hopefully arachnid monophyly too - but this goes beyond the scope of the present study. We refer the reader to Dunlop [104] for a review of possible chelicerate origins and to our character statements (Additional file 5), in which paleontological information is discussed where relevant.

## Conclusions

Previous studies combining ribosomal sequences and morphology recovered topologies similar to those morphological analyses which yielded taxa such as Haplocnemata and Acari. Comparing the results of the methods for molecular homology assessment employed here, we conclude that the apparent stability of the clades noted above in total evidence analyses is better explained as the byproduct of the way the molecular homology was established using the instrumentalist approach implemented in POY. Constraining the analysis by *a priori *homology assessment is defended as a way of maintaining the severity of the test when adding new data to the analysis. Although the strength of this methodology is that it retains phylogenetic information from regions usually discarded in an exclusively static homology framework, it still has the inconvenience of being uninformative on the effect of alignment ambiguity on resampling methods of clade support estimation. Finally, the most notable result of our analysis is further evidence for a strong molecular signal supporting Solifugae + Acariformes. Morphological apomorphies for this clade - for which we here adopt the name Poecilophysidea - include reduction of the proximal cheliceral podomere, medial abutting of the leg coxae, loss of sperm nuclear membrane, and presence of differentiated germinative and secretory regions in the testis delivering their products into a common lumen. This last character in particular has been widely overlooked in recent morphological studies concerning chelicerate phylogeny.

## Authors' contributions

ARP designed this study, carried out the molecular alignment employing the rRNA secondary structure, conducted Bayesian and POY analyses, participated in the scoring and elaboration of morphological matrices and drafted the manuscript. CEFR participated in the data acquisition, in study design and helped to draft the manuscript. JAD elaborated the morphological character statements, participated in the scoring of morphological matrices, wrote substantial portion of the discussion section and helped to draft the manuscript. All authors read and approved its final version.

## Supplementary Material

Additional file 1 **Sampling data and taxonomy**. The table indicate the taxonomy and collection information for the species newly sequenced and associated accession number for the MZUSP collection. Click here for file

Additional file 2 **Secondary structure alignments**. Two FASTA files containing the secondary structure alignments of 18 S and 28 S are provided, along with two notations marks, a pairing mask with signals such "( )", "{ }", and "< >" for paired sites, indicating the pair members; ".", for unpaired sites and "*", for regions of ambiguous alignment. The other mask indicates secondary structures as labeled in the Fig. 2, 3. Click here for file

Additional file 3 **Monitoring the convergence of MCMC in Bayesian analyses**. Plotings of the LnL of the stationary phase of each one of the models along with a comparison of parameters values obtained from the two independent runs using Gelman's statistic [105] are provided. Click here for file

Additional file 4 **POY imputs.** Fasta files including all the regions of ambiguous alignment are provided. Click here for file

Additional file 5 **Morphological characters statements**. The file provided include statements of the 178 morphological characters used in the combined phylogenetic analyses and gathered along the present study. Click here for file

Additional file 6 **Morphological datasets**. Two data matrices are provided, that produced by scoring character statements gathered along the present study (Matrix A) and those enunciated by Shultz [22] (Matrix B). Click here for file

Additional file 7 **Tables with ILD metrics values for analysis employing direct optimization**. The file contains ILD metrics values for the standard and constrained analysis of molecular data alone, and combined analysis of molecular data and morphological data matrices A and B. Click here for file
