# Supplementary material for: Phylogenetic position of the acariform mites: sensitivity to homology assessment under total evidence
Source: BMC Evol Biol. 2010 Aug 2;10:235. doi: 10.1186/1471-2148-10-235 (PMC2933639; doi:10.1186/1471-2148-10-235)
Supplement: Additional file 1 — Sampling data and taxonomy. The table indicate the taxonomy and collection information for the species newly sequenced and associated accession number for the MZUSP collection. [file 1471-2148-10-235-S1.DOC]

| **Taxa** | **Locality** | **Coordinates** | **Date** | **Substrate** | **Collector** | **Vouchering** |
| --- | --- | --- | --- | --- | --- | --- |
| **Order Amblypygi** |  |  |  |  |  |  |
| *Charinus* *montanus* | Santa Teresa city, ES, Brazil | 19º 55’S, 40º 35’W | 15-19.X.2003 | Litter | A. Giupponi | MZSP 32462 |
| **Order Araneae** |  |  |  |  |  |  |
| Corinnidae | Campus Universidade de São Paulo,São Paulo, SP, Brazil. | 23º 34’S, 64º 44’W | 20. X. 2008 | Soil. | A. Pepato | MZSP 32463 |
| Tetragnathidae | Campus Universitade de Santiago de Compostela, Lugo, Spain | 42˚ 59’N, 7˚ 33’W | 18. VIII. 2008 | Litter | A. Pepato | MZSP 32464 |
| Pholcidae | Campus Universidade de São Paulo,São Paulo, SP, Brazil. | 23º 34’S, 64º 44’W | 20. X. 2008 | House inhabiting | A. Pepato | MZSP 32465 |
| **Order Palpigradi** |  |  |  |  |  |  |
| *Eukoenenia* sp. | Mina Várzea do Lopes Itabirito, MG, Brazil. | 20º 25’S, 43º 55’W | 18-25. IV 2007 | Cave soil | R. Andrade | MZSP 32466 |
| **Superorder Acariformes** |  |  |  |  |  |  |
| **Order Sarcoptiformes** |  |  |  |  |  |  |
| **Suborder Oribatida** |  |  |  |  |  |  |
| **Supercohort Desmonomatides** |  |  |  |  |  |  |
| **Cohort Brachypylina** |  |  |  |  |  |  |
| **Family Tetracondylidae** |  |  |  |  |  |  |
| *Pseudotocepheus* *septemtuberculata* Balogh & Mahunka, 1978 | Campus Universidade de São Paulo,São Paulo, SP, Brazil. | 23º 34’S, 64º 44’W | 02. IX. 2007. | Litter | A. Pepato | MZSPAC 00002 |

| **Taxa** | **Locality** | **Coordinates** | **Date** | **Substrate** | **Collector** | **Vouchering** |
| --- | --- | --- | --- | --- | --- | --- |
| **Family Haplozetidae** |  |  |  |  |  |  |
| *Rostrozetes* *ovulum* (Berlese, 1908) | Campus Universidade de São Paulo,São Paulo, SP, Brazil. | 23º 34’S, 64º 44’W | 02. IX. 2007 | Litter | A. Pepato | MZSPAC 00003 |
| **Family Oppiidae** |  |  |  |  |  |  |
| *Aeroppia* sp. | Nova Granada, SP, Brasil | 20° 32'S, 49° 14'W | 23.V.2007 | Plant leaves | A. Pepato | MZSPAC 00005 |
| **Family Selenoribatidae** |  |  |  |  |  |  |
| *Schusteria littoria* Grandjean, 1968 | Araçá mangrove, São Sebastião, São Paulo, Brazil | 23˚ 48’S, 45˚ 24’W | 25 II 2008 | Green algae | A. Pepato | MZSPAC 00006 |
| **Cohort Nothrina** |  |  |  |  |  |  |
| **Family Trhypochthoniidae** |  |  |  |  |  |  |
| *Afronothrus* sp. | Campus Universidade de São Paulo,São Paulo, SP, Brazil. | 23º 34’S, 64º 44’W | 02. IX. 2007 | Litter | A. Pepato | MZSPAC 00004 |
| **Cohort Astigmata** |  |  |  |  |  |  |
| **Family Acaridae** |  |  |  |  |  |  |
| *Sancassania* sp | Butantã Institute, São Paulo Brazil. | 23˚ 34’S, 46˚ 43’W | 05. X. 2007 | Associated to *Gymnophiona*  in captivity | A. Pepato | MZSPAC 00017 |
| *Rhizoglyphus* sp. | Carajás, Pará, Brazil. | 2˚ 56’S, 51˚ 51’W | 23. VIII- 02. IX. 2007 | Cave soil | R. Andrade | MZSPAC 00018 |

| **Taxa** | **Locality** | **Coordinates** | **Date** | **Substrate** | **Collector** | **Vouchering** |
| --- | --- | --- | --- | --- | --- | --- |
| **Suborder Endeostigmata** |  |  |  |  |  |  |
| **Cohort Alycina** |  |  |  |  |  |  |
| **Family Alycidae** |  |  |  |  |  |  |
| *Bimichaelia* sp. | Cidade Universitária,São Paulo, SP, Brazil. | 23º 34’S, 64º 44’W | 03. IX. 2007 | Litter | A. Pepato | MZSPAC 00001 |
| **Order Trombidiformes** |  |  |  |  |  |  |
| **Suborder Prostigmata** |  |  |  |  |  |  |
| **Supercohort Eupodides** |  |  |  |  |  |  |
| **Family Bdellidae** |  |  |  |  |  |  |
| *Bdellodes* sp. | Mina de Congo Soco, Barão de Cocais, MG, Brazil | 19º 56’S, 43º 28’W | 22-24. XI. 1997 | Cave soil | R. Andrade | MZSPAC 00019 |
| *Spinibdella* sp. | Cidade Universitária,São Paulo, SP, Brazil. | 23º 34’S, 64º 44’W | 06. X. 2008 | Moss | A. Pepato | MZSPAC 00029 |
| **Family Rhagidiidae** |  |  |  |  |  |  |
| *Poecilophysis* sp. | Campus da Universitade de Santiago de Compostela, Lugo, Spain | 42˚ 59’N, 7˚ 33’W | 18. VIII. 2008 | Litter | A. Pepato | MZSPAC 00021 |
| Rhagidiidae sp. | Mina Várzea do Lopes Itabirito, MG, Brazil | 20º 25’S, 43º 55’W | 18-25. Iv. 07 | Cave soil | R. Andrade | MZSPAC 00014 |
| **Family Halacaridae** |  |  |  |  |  |  |
| *Halacarus* sp. | Lázaro Beach, Ubatuba, São Paulo, Brazil. | 23^o^30’S, 45^o^08’W | XI. 2005 | Algae | A. Pepato | MZSPAC 00011 |
| *Rhombognathus* *levigatoides* Pepato & Rocha, 2007 | Pitangueiras Beach, São Sebastião, São Paulo, Brazil. | 23º 49’S 45º 25’W | 07. XI. 2006 | Algae | A. Pepato | MZSPAC 00012 |
| **Family Eupodidae** |  |  |  |  |  |  |
| *Eupodes* sp. | Cidade Universitária,São Paulo, SP, Brazil. | 23º 34’S, 64º 44’W | 06. X. 2008 | Moss | A. Pepato | MZSPAC 00026 |
| **Supercohort Eleutherengonides** |  |  |  |  |  |  |
| **Cohort Raphignathina** |  |  |  |  |  |  |
| **Family Tenuipalpidae** |  |  |  |  |  |  |
| *Tenuipalpus* *heveae* Baker, 1945 | Laboratório de Acarologia-IBILCE-UNESP, São José do Rio Preto, SP, Brazil. | 20º 47’S, 49º 21’W | 23.V.2007 | Cultured individuals | R. Feres. | MZSPAC 00031 |
| **Family Tetranychidae** |  |  |  |  |  |  |
| *Tetranychus* sp. | Cidade Universitária,São Paulo, SP, Brazil. | 23º 34’S, 64º 44’W | 22. IX. 2007 | Leaves of a papaya tree | A. Pepato | MZSPAC 00030 |
| *Sonotetranychus* sp. | Cidade Universitária,São Paulo, SP, Brazil. | 23º 34’S, 64º 44’W | 15. IX. 2008; | Plant leaves | A. Pepato | MZSPAC 00032 |
| **Family Cheyletidae** |  |  |  |  |  |  |
| *Oudemansicheyla* sp. | Cidade Universitária,São Paulo, SP, Brazil. | 23º 34’S, 64º 44’W | 15. IX. 2008 | Plant leaves | A. Pepato | MZSPAC 00023 |
| *Cheletomimus* (*Hemicheyletia*) *wellsi* (Baker, 1949a) | Cidade Universitária,São Paulo, SP, Brazil. | 23º 34’S, 64º 44’W | 15. IX. 2008 | Plant leaves | A. Pepato | MZSPAC 00024 |
| **Family Stigmaeidae** |  |  |  |  |  |  |
| *Agistemus* sp. | Cidade Universitária,São Paulo, SP, Brazil. | 23º 34’S, 64º 44’W | 06. X. 2008 | Moss | A. Pepato | MZSPAC 00027 |

| **Taxa** | **Locality** | **Coordinates** | **Date** | **Substrate** | **Collector** | **Vouchering** |
| --- | --- | --- | --- | --- | --- | --- |
| **Supercohort Anystides** |  |  |  |  |  |  |
| **Cohort Anystina** |  |  |  |  |  |  |
| **Family Paratydeidae** |  |  |  |  |  |  |
| *Scolotydaeus* *corticicola* Flechtmann, 1992 | Cidade Universitária,São Paulo, SP, Brazil. | 23º 34’S, 64º 44’W | 20. X. 2008 | Bark | A. Pepato | MZSPAC 00028 |
| **Family Anystidae** |  |  |  |  |  |  |
| *Erythracarus* sp. | Mina de Congo Soco, Barão de Cocais, MG, Brazil | 19º 56’S, 43º 28’W | 22-24. XI. 1997 | Cave soil | R. Andrade | MZSPAC 00020 |
| *Anystis* sp. | Campus da Universitade de Santiago de Compostela, Lugo, Spain | 42˚ 59’N, 7˚ 33’W | 18. VIII. 2008 | Litter | A. Pepato | MZSPAC 00022 |
| **Cohort Parasitengonina** |  |  |  |  |  |  |
| **Family Trombiculidae** |  |  |  |  |  |  |
| *Hoffmaniella* (?)* | Segredo Beach, São Sebastião, São Paulo, Brazil. | 23˚ 49’S, 45˚ 25’W | 21.III. 2007 | Rockshore seaweed | A. Pepato | MZSPAC 00015 |
| **Family Microtrombidiidae** |  |  |  |  |  |  |
| *Microtrombidium* sp | Mina Várzea do Lopes Itabirito, MG, Brazil | 20º 25’S, 43º 55’W | 18-25. IV. 07 | Cave soil | R. Andrade | MZSPAC 00013 |
| **Family Smaridiidae** |  |  |  |  |  |  |
| Smaridiidae sp. | Cidade Universitária,São Paulo, SP, Brazil. | 23º 34’S, 64º 44’W | 22. IX. 2008 | Litter | A. Pepato | MZSPAC 00025 |

| **Taxa** | **Locality** | **Coordinates** | **Date** | **Substrate** | **Collector** | **Vouchering** |
| --- | --- | --- | --- | --- | --- | --- |
| **Family Erythraeidae** |  |  |  |  |  |  |
| *Leptus* sp. | Cidade Universitária,São Paulo, SP, Brazil. | 23º 34’S, 64º 44’W | 05. IX. 2007 | Litter | A. Pepato | MZSPAC 00016 |
| **Family Arrenuridae** |  |  |  |  |  |  |
| *Arrenurus* sp. | Tramandaí River, Imbé, Rio Grande do Sul, Brazil. | 29˚ 58’S, 50˚ 09’W | 04. X. 2006 | Aquatic weeds | A. Pepato | MZSPAC 00010 |
| **Family Hydrachnidae** |  |  |  |  |  |  |
| *Hydrachna* sp. | Parque Ecológico do Tietê, São Paulo, SP, Brazil. | 23˚ 29’S, 46˚ 31’W | 05. III. 2007 | On aquatic hemiptera | A. Pepato | MZSPAC 00009 |
| **Family Unionicolidae** |  |  |  |  |  |  |
| *Recifella* sp. | Ribeira do Iguape River, Iguape, São Paulo, Brazil. | 24˚ 39’S, 46˚ 31’W | 17.IV.2007 | Aquatic weeds | A. Pepato | MZSPAC 00008 |
| **Family Limnesiidae** |  |  |  |  |  |  |
| *Limnesia* sp. | Billings Reservoir, São Paulo, SP, Brazil. | 23˚ 49’S, 46˚ 37’W | 18. II. 2008 | Aquatic weeds | A. Pepato | MZSPAC 00007 |

*, The Trombiculidae taxonomy relies largely on larvae. A larvae clearly associated to the adult individuals employed in this study belongs to the genus *Hoffmaniella,* but without a complete ontogenetic study of the species it is hard to assign the specimens to this genus by sure.
