# Supplementary material for: Phylogenetic position of the acariform mites: sensitivity to homology assessment under total evidence
Source: BMC Evol Biol. 2010 Aug 2;10:235. doi: 10.1186/1471-2148-10-235 (PMC2933639; doi:10.1186/1471-2148-10-235)
Supplement: Additional file 7 — Tables with ILD metrics values for analysis employing direct optimization. The file contains ILD metrics values for the standard and constrained analysis of molecular data alone, and combined analysis of molecular data and morphological data matrices A and B. [file 1471-2148-10-235-S7.DOC]

**Incongruence Length Difference summaries**

**Table 1-** ILD metrics for the standard and constrained analysis of molecular data

| Cost regime | POY standard analysis | | | | POY constrained analysis | | | |
| --- | --- | --- | --- | --- | --- | --- | --- | --- |
|  | Combined Tree Length | SSU Tree Length | LSU Tree Length | ILD | Combined Tree Length | SSU Tree Length | LSU Tree Length | ILD |
| 1:1:1 | 9998 | 7544 | 2338 | 0,0116 | 10095 | 7616 | 2359 | 0,0119 |
| 1:1:2 | 15857 | 11931 | 3699 | 0,0143 | 12945 | 9620 | 3120 | 0,0158 |
| 1:2:1 | 15200 | 11430 | 3567 | 0,0134 | 13335 | 9891 | 3248 | 0,0147 |
| 2:1:1 | 11711 | 8702 | 2789 | 0,0188 | 11637 | 8636 | 2818 | 0,0157 |
| 2:1:2 | 18805 | 13960 | 4338 | 0,0270 | 15709 | 11466 | 3815 | 0,0272 |
| 2:2:1 | 18361 | 13567 | 4325 | 0,0255 | 16231 | 11791 | 4031 | 0,0252 |
| 4:1:1 | 14427 | 10596 | 3308 | 0,0363 | 14288 | 10391 | 3467 | 0,0301 |
| 4:1:2 | 23903 | 17564 | 5144 | 0,0500 | 20824 | 14913 | 4960 | 0,0457 |
| 4:2:1 | 23633 | 17234 | 5283 | 0,0472 | 21355 | 15210 | 5235 | 0,0426 |

**Table 2-** ILD metrics for combined analyses employing Shultz [22] character definitions (matrix B). Note that values for the morphological matrix were corrected according to the weights assigned to keep an approximate correspondence to the relative weight of the morphological data in the 1:1:1 cost regime.

| Cost  regime | POY standard analysis | | | | POY constrained analysis | | | |
| --- | --- | --- | --- | --- | --- | --- | --- | --- |
|  | Morph. | ∑ SSU-LSU Length | Combined Tree Length | ILD | Morph | ∑ SSU-LSU Length | Combined Tree Length | ILD |
| 1:1:1 | 420,0 | 9882 | 10491,0 | 0,0180 | 420,0 | 9975 | 10587,0 | 0,0181 |
| 1:1:2 | 666,1 | 15630 | 16657,6 | 0,0217 | 538,6 | 12740 | 13590,9 | 0,0230 |
| 1:2:1 | 638,5 | 14997 | 15923,0 | 0,0181 | 554,8 | 13139 | 13976,0 | 0,0202 |
| 2:1:1 | 492,0 | 11491 | 12304,0 | 0,0261 | 484,2 | 11454 | 12229,0 | 0,0238 |
| 2:1:2 | 790,0 | 18298 | 19801,4 | 0,0360 | 653,6 | 15281 | 16530,6 | 0,0361 |
| 2:2:1 | 771,3 | 17892 | 19265,0 | 0,0312 | 675,3 | 15822 | 17031,2 | 0,0313 |
| 4:1:1 | 606,1 | 13904 | 15194,0 | 0,0450 | 594,4 | 13858 | 15011,2 | 0,0372 |
| 4:1:2 | 1004,1 | 22708 | 25226,0 | 0,0600 | 866,4 | 19873 | 21970,4 | 0,0560 |
| 4:2:1 | 992,8 | 22517 | 24981,0 | 0,0589 | 888,5 | 20445 | 22488,1 | 0,0513 |

**Table 3**. ILD metrics for combined analyses employing the character definitions presented herein (Matrix A). Note that values for the morphological matrix were corrected according to the weights assigned to keep an approximate correspondence to the relative weight of the morphological data in the 1:1:1 cost regime.

| Cost  regime | POY standard analysis | | | | POY constrained analysis | | | |
| --- | --- | --- | --- | --- | --- | --- | --- | --- |
|  | Morph. | ∑ SSU-LSU Length | Combined Tree Length | ILD | Morph | ∑ SSU-LSU Length | Combined Tree Length | ILD |
| 1:1:1 | 385,0 | 9882 | 10451,0 | 0,0176 | 385,0 | 9975 | 10548,0 | 0,0178 |
| 1:1:2 | 610,6 | 15630 | 16600,8 | 0,0217 | 493,7 | 12740 | 13538,3 | 0,0225 |
| 1:2:1 | 585,3 | 14997 | 15865,0 | 0,0178 | 508,6 | 13139 | 13920,1 | 0,0196 |
| 2:1:1 | 451,0 | 11491 | 12257,4 | 0,0257 | 443,8 | 11454 | 12177,4 | 0,0230 |
| 2:1:2 | 724,1 | 18298 | 19720,4 | 0,0354 | 599,1 | 15281 | 16483,0 | 0,0366 |
| 2:2:1 | 707,0 | 17892 | 19198,4 | 0,0312 | 619,0 | 15822 | 16986,0 | 0,0321 |
| 4:1:1 | 555,6 | 13904 | 15122,6 | 0,0438 | 544,9 | 13858 | 14966,2 | 0,0376 |
| 4:1:2 | 920,4 | 22708 | 25123,6 | 0,0595 | 794,2 | 19873 | 21917,2 | 0,0570 |
| 4:2:1 | 910,1 | 22517 | 24873,6 | 0,0582 | 814,4 | 20445 | 22438,4 | 0,0525 |
